# Repeated Internal Validation and Interpretation of Models Predicting Surgery Documented at a Study Institution Within 90 Days After Acute Knee Trauma: A Secondary Analysis

**DOI:** 10.3390/medicina62101821

**Published:** 2026-09-22

**Authors:** Seongho Hwang, Joungeun Lee, Won-Kee Choi, Young Woo Seo, Sang Gyu Kwak

**Affiliations:** 1 Department of Emergency Medicine, Daegu Catholic University Hospital, Daegu 42472, Republic of Korea; mt12xt@dcmc.co.kr (S.H.); zaiaz12815@dcmc.co.kr (J.L.); 2 Department of Orthopaedic Surgery, Daegu Catholic University School of Medicine, Daegu 42472, Republic of Korea; cwk10090@cu.ac.kr; 3 Department of Emergency Medicine, Daegu Catholic University School of Medicine, Daegu 42472, Republic of Korea; 4 Department of Medical Statistics, Daegu Catholic University School of Medicine, Daegu 42472, Republic of Korea

**Keywords:** acute knee trauma, emergency department, gradient boosting, internal validation, machine learning, permutation importance, random forest, surgical intervention

## Abstract

*Background and Objectives:* A previous study using this cohort evaluated threshold-dependent performance and clinical decision utility for predicting surgery after acute knee trauma. This secondary analysis assessed the stability of discrimination, probabilistic accuracy, calibration, model ranking, and predictor importance under repeated internal validation. *Materials and Methods:* We analyzed 905 adults with acute knee trauma, including 163 (18.0%) who had a qualifying surgical procedure documented at the study institution within 90 days of the index emergency department visit. Logistic regression, random forest, and gradient-boosted trees were evaluated using 50 repetitions of stratified five-fold cross-validation with identical folds across models. Each patient’s 50 out-of-fold predictions were averaged. Confidence intervals and paired model differences were estimated using 2000 patient-level stratified bootstrap resamples. Random-forest predictor importance was evaluated by validation-fold permutation. *Results:* Logistic regression and random forest had AUROCs of 0.762 and 0.757, AUPRCs of 0.430 and 0.443, and Brier scores of 0.125 and 0.123, respectively. Paired comparisons between these two models did not indicate statistically significant differences in any of the three metrics. Random forest had a higher AUPRC and lower Brier score than gradient-boosted trees. Calibration-in-the-large was close to 0 for all three models, whereas the calibration slope was compatible with 1 for logistic regression and random forest but was below 1 for gradient-boosted trees. Knee swelling, limited range of motion, and injury mechanism were the most influential random-forest predictors. *Conclusions:* Repeated internal validation did not identify a uniformly superior model. Logistic regression most often ranked first for AUROC, whereas random forest most often ranked first for AUPRC and Brier score; however, paired comparisons did not identify significant differences between these two models. External validation is required before clinical use.

## 1. Introduction

Acute knee trauma encompasses a heterogeneous spectrum of conditions, including fractures, ligamentous injuries, meniscal lesions, patellar instability or dislocation, and other osteochondral or soft-tissue injuries [1]. These conditions differ substantially in their indications and timelines for operative treatment. Displaced fractures and unstable injuries may require early surgery, whereas many ligamentous, meniscal, and patellar injuries are initially managed with analgesia, activity modification, immobilization or bracing when appropriate, rehabilitation, and specialist reassessment [2]. Surgery may therefore occur urgently, after recovery of swelling and range of motion, or following unsuccessful non-operative management, depending on the injury type and patient-specific factors. Consequently, surgical intervention within 90 days represents a heterogeneous treatment pathway rather than a homogeneous biological outcome.

Prediction models can combine information available at presentation to estimate the probability of a subsequent clinical event. Evaluation of such models should extend beyond a single measure of discrimination. Precision–recall performance is informative when the outcome is relatively uncommon, calibration assesses agreement between predicted and observed risks, and interpretation methods can help identify the recorded features contributing to individual predictions [3,4,5,6,7,8]. These complementary assessments are particularly relevant when models are intended to support early clinical evaluation rather than replace clinical judgment.

Machine learning methods have increasingly been investigated in orthopedic research, including applications for predicting revision surgery, recurrent injury, and subsequent arthroplasty [9,10,11,12]. However, estimated performance may depend on the data partition, model specification, and validation procedure. A model that performs favorably in one randomly selected holdout sample may yield different estimates in another, particularly in a moderate-sized, single-center cohort. Transparent assessment of this analytic variability is therefore important when interpreting internally validated prediction models [13,14,15,16].

The cohort and endpoint examined in this secondary analysis are identical to those of our previous publication [17]; in both studies, the endpoint was a qualifying surgical procedure documented at the study institution within 90 days of the index emergency department (ED) visit. Whereas the previous study focused on threshold-specific performance, decision-curve analysis, temporal validation, and risk stratification, the present study examined the robustness of logistic regression, random forest, and gradient-boosted trees across 50 repetitions of stratified five-fold cross-validation. Specifically, we evaluated the stability of discrimination, precision–recall performance, probabilistic accuracy, calibration, model ranking, and predictor importance, together with paired model differences and validation-fold permutation importance. The present study was therefore designed as an exploratory robustness analysis rather than independent model development or validation in a new cohort.

## 2. Methods

### 2.1. Study Design and Setting

We performed a single-center retrospective secondary analysis of electronic medical records from the ED of Daegu Catholic University Medical Center. Eligible visits occurred between 1 January 2016 and 30 September 2025. The present secondary analysis was not prospectively registered, and no publicly accessible time-stamped statistical analysis protocol was available. The resampling scheme, model specifications, preprocessing procedures, and evaluation metrics used in the revised analysis were fixed before the repeated-validation analysis was conducted and were not selected by examining its validation results. The study was reviewed and approved by the Institutional Review Board of Daegu Catholic University Medical Center (IRB No. DCUMC 2026-01-003), which waived the requirement for informed consent because the study used de-identified retrospective data. The study was conducted in accordance with the Declaration of Helsinki and reported with reference to the TRIPOD + AI statement [13].

The cohort and endpoint examined in the present study completely overlap with those of our previous publication [17]; in both studies, the endpoint was a qualifying surgical procedure documented at the study institution within 90 days of the index ED visit. The separate IRB approval is reported solely to document ethical oversight and should not be interpreted as establishing scientific independence from the previous study. The previous publication focused primarily on threshold-specific performance, decision-curve analysis, temporal validation, and risk stratification, whereas the present secondary analysis evaluates repeated-resampling variability, paired model differences, quantitative calibration, model-rank stability, and validation-fold predictor importance. The present study is therefore described as an exploratory robustness analysis rather than independent model development or validation in a new cohort. No previously published figures or tables were reused. A detailed comparison of the analyses and results reported in the previous publication and the present secondary analysis is provided in Supplementary Table S1.

### 2.2. Study Population and Eligibility Criteria

We screened adult patients aged ≥18 years who visited the ED during the study period with an acute knee injury identified using International Classification of Diseases, 10th Revision codes S80–S89. For patients with more than one eligible ED visit, only the first visit was included.

Patients were excluded if they had chronic or degenerative knee disease, infectious knee disease, polytrauma involving injuries to multiple body regions, missing data for at least one candidate predictor, or insufficient institutional follow-up to ascertain whether a qualifying surgical procedure was documented within 90 days. In the de-identified dataset available for this secondary analysis, exclusions due to missing predictor data and insufficient follow-up were retained only as a combined category. Consequently, the two exclusion mechanisms could not be quantified separately, and individual-level baseline data for the excluded patients were unavailable.

Only patients whose 90-day outcome status could be ascertained from the institutional electronic medical records were included. Patients without sufficient follow-up records to determine whether surgical intervention occurred within 90 days were excluded rather than classified as not having undergone surgery. Among the included patients, those with available follow-up records but no documented surgical intervention within 90 days were classified as the non-surgical group.

### 2.3. Outcome Definition

The study outcome was a qualifying surgical procedure documented as having been performed at the study institution within 90 days after the index ED visit. Surgical procedures were identified from institutional electronic medical records using operative records and procedure codes. The 90-day interval was selected to capture both early and delayed operative treatment following the index presentation; however, the available data did not permit systematic identification or exclusion of an intervening traumatic event.

The recorded procedure categories included arthroscopy, open reduction and internal fixation, external fixation, meniscus repair, partial meniscectomy, anterior or posterior cruciate ligament reconstruction, collateral or multiligament repair, multiligament reconstruction, arthroplasty, and staged procedures. The categories were mutually exclusive in the analytic dataset. No cases were recorded under separate categories for patellar procedures or extensor-mechanism repair. A separate structured variable for the injury-specific operative diagnosis was unavailable; therefore, operative diagnoses were not retrospectively inferred from the procedure categories.

Patients with a qualifying surgical procedure documented at the study institution within 90 days were classified as having the study outcome. Patients with sufficient institutional follow-up but no qualifying procedure documented at the study institution during this period were classified as not having the outcome. Surgical procedures performed at other institutions could not be identified from the available data. Consequently, the endpoint represents surgery documented at the study institution within 90 days rather than all surgery performed within 90 days.

### 2.4. Predictor Variables

Candidate predictors were selected a priori based on their availability during the initial ED assessment and their potential clinical relevance to the likelihood of subsequent surgical intervention. The predictors comprised age, sex, body mass index (BMI), injury mechanism, numeric rating scale (NRS) pain score, knee swelling, limitation of range of motion (ROM), Korean Triage and Acuity Scale (KTAS) level, and abnormal findings on plain radiography.

Age, BMI, and NRS pain score were analyzed as continuous variables. Sex, injury mechanism, knee swelling, ROM limitation, and abnormal radiographic findings were represented using their prespecified categorical or binary coding. KTAS was analyzed according to the levels recorded at ED presentation. Predictor information was extracted exclusively from records generated during the index ED visit; information documented after the initial assessment was not used for prediction.

Injury mechanism was classified from the ED documentation as fall, sports injury, twisting injury, traffic accident, or direct blow and was represented using indicator variables. KTAS was recorded on the five-level Korean Triage and Acuity Scale (1, resuscitation; 2, emergent; 3, urgent; 4, less urgent; and 5, non-urgent), with lower levels indicating greater urgency, and was analyzed as an ordinal numeric predictor.

Knee swelling was coded as present when the initial ED physical examination explicitly documented swelling, effusion, or joint effusion of the injured knee. ROM limitation was coded as present when the initial ED record explicitly documented limited or decreased ROM, restricted flexion or extension, pain-limited motion, or mechanical block. Both variables were recorded as binary findings based on routine clinical documentation and were not derived from quantitative circumference measurements, an effusion grading scale, or goniometric thresholds. Predictor data were abstracted by two investigators (Y.W.S. and W.-K.C.), and disagreements were resolved by consensus. Because the investigators’ initial individual ratings before consensus were not retained, interobserver agreement could not be calculated. An abnormal radiographic finding was defined as an acute traumatic abnormality documented in the formal radiology report or ED record, including fracture, dislocation, or another acute osseous or joint abnormality. Detailed definitions and coding of all predictor variables are provided in Supplementary Table S2.

### 2.5. Data Preprocessing

The analysis was restricted to 905 patients with complete data for all nine candidate predictors and an ascertainable study outcome. Accordingly, the proportion of missingness in the final analytic cohort was 0% for each candidate predictor: age, sex, BMI, injury mechanism, pain score, knee swelling, ROM limitation, KTAS level, and abnormal radiographic findings. However, the 128 patients excluded because of missing predictor data and/or insufficient institutional follow-up were retained only as a combined exclusion category in the de-identified dataset. Because individual-level records and predictor-specific missingness indicators for these excluded patients were unavailable, the proportion of missingness for each predictor among all screened patients could not be reconstructed.

Multiple imputation was considered during the revision but was not performed because the individual-level records and predictor-specific missingness patterns of the excluded patients were unavailable. Moreover, imputation of missing predictor values would not resolve the absence of outcome ascertainment among patients with insufficient institutional follow-up. The analysis therefore remained restricted to patients with complete predictor data and an ascertainable outcome.

Within each training fold, continuous predictors were standardized and categorical predictors were one-hot encoded. All preprocessing parameters were estimated exclusively from the training fold and applied unchanged to the corresponding validation fold. Binary predictors were represented as 0/1 indicators.

The three models were evaluated using 50 repetitions of stratified five-fold cross-validation. Identical fold assignments were used for all three models within each repetition to permit paired comparison. In each repetition, every patient was included in a validation fold exactly once and received an out-of-fold predicted probability from each model. Thus, each patient had 50 out-of-fold predicted probabilities per model, which were averaged to obtain the patient-level predicted probabilities used for the primary performance and calibration analyses. No separate held-out test set was used.

### 2.6. Model Development

Three classification algorithms were evaluated: logistic regression, random forest, and gradient-boosted trees. Logistic regression was fitted using L2 regularization. Random forest was fitted using 500 trees, a maximum tree depth of 10, a minimum of four observations per terminal leaf, and the square root of the available features considered at each split. The gradient-boosted tree model used 300 estimators, a learning rate of 0.03, a maximum tree depth of 3, a minimum of four observations per terminal leaf, row subsampling of 0.9, and feature subsampling of 0.9. These settings were fixed before execution of the revised analysis, and no data-driven hyperparameter search was performed. Complete model specifications are provided in Supplementary Table S3.

The available sample size was determined by the fixed retrospective cohort, and no formal prospective sample-size calculation was performed. Sample adequacy was not assessed using a fixed events-per-variable threshold because such a rule does not adequately characterize the complexity or sample requirements of ensemble tree models. Instead, the revised analysis was restricted to nine clinically selected presentation-time predictors, and model complexity was constrained using regularization, maximum tree depth, minimum leaf size, and fixed hyperparameters [18,19]. In each repetition of stratified five-fold cross-validation, approximately 724 patients, including approximately 130 patients with the outcome, were used for model fitting, and approximately 181 patients, including 32–33 patients with the outcome, were used for validation. These measures were intended to reduce overfitting and quantify internal variability but do not establish adequacy for definitive model development or clinical implementation.

No class weighting, over-sampling, or under-sampling was applied to any model. The primary objective was to estimate outcome probabilities under the observed event prevalence of 18.0%, and calibration and Brier score were central evaluation measures. Class weighting changes the relative contribution of events and non-events to the training loss and may alter the calibration of predicted probabilities. We therefore used the same unweighted training approach for all three models. The effect of outcome imbalance on model evaluation was addressed by reporting precision–recall curve (AUPRC) together with receiver operating characteristic curve (AUROC), Brier score, and calibration measures.

### 2.7. Model Performance Evaluation

Discrimination was evaluated using the AUROC, precision–recall performance using the AUPRC, and overall probabilistic accuracy using the Brier score, with lower Brier scores indicating better accuracy. Within each cross-validation repetition, performance metrics were calculated by pooling the out-of-fold predictions from the five validation folds. The distributions of these metrics across the 50 repetitions were used to describe resampling variability and model-rank stability.

Primary patient-level performance estimates were calculated using each patient’s average out-of-fold predicted probability across the 50 repetitions. Ninety-five percent confidence intervals for the AUROC, AUPRC, and Brier score were estimated using 2000 stratified patient-level bootstrap resamples and the 2.5th and 97.5th percentiles of the bootstrap distributions. Because all three models generated predictions for the same patients, pairwise differences in each performance measure were calculated within the same bootstrap resamples, thereby preserving the paired structure. Two-sided bootstrap *p* values were calculated as twice the smaller proportion of bootstrap differences falling on either side of zero. The paired comparison results are provided in Supplementary Table S4.

Calibration was evaluated quantitatively and graphically using each patient’s average out-of-fold predicted probability across the 50 cross-validation repetitions. Calibration-in-the-large was estimated using a logistic model with the logit of the average out-of-fold predicted probability included as an offset, thereby fixing its coefficient at 1. The calibration slope was estimated from the logistic recalibration model $logit[Pr(Y=1)]\;=\;\alpha +\beta logit(\hat{p})$, where $\hat{p}$ denotes the average out-of-fold predicted probability and β denotes the calibration slope. Ideal calibration corresponds to a calibration-in-the-large value of 0 and a calibration slope of 1. Ninety-five percent confidence intervals were estimated using 2000 stratified patient-level bootstrap resamples. For graphical assessment, patients were divided into 10 equal-frequency groups separately for each model, and the mean predicted probability was compared with the observed proportion of patients with the study outcome in each group. The underlying group-level calibration results are provided in Supplementary Table S5.

### 2.8. Methods for Model Interpretation

Predictor importance was evaluated for the random forest model using validation-fold permutation importance. Random forest was selected as the targeted interpretability model before execution of the revised analysis because it was the principal nonlinear model for which predictor-attribution patterns had been examined in our previous study [17]. Its selection was not based on statistically significant superiority over logistic regression. The purpose of this analysis was to assess the robustness of predictor-importance patterns for a representative nonlinear model rather than to compare attribution measures across all three algorithms.

Permutation importance was calculated in 50 validation folds obtained from 10 repetitions of stratified five-fold cross-validation. Within each validation fold, the values of each original predictor were randomly permuted three times while the fitted random forest model and all other predictors were held unchanged. Importance was quantified as the decrease in validation-fold AUROC and AUPRC after permutation relative to the unpermuted predictions. The primary ranking was based on the mean decrease in AUPRC across validation folds and permutations. Positive values indicate deterioration in predictive performance after permutation, whereas values near or below zero indicate little measurable contribution.

Permutation importance is conditional on the fitted random forest model and the distribution and correlation structure of the predictors. It does not indicate the direction of a predictor’s association, establish an independent effect, or imply causality. Because the analysis was restricted to random forest, the resulting importance pattern should not be assumed to apply to logistic regression or gradient-boosted trees.

### 2.9. Statistical Analysis

Continuous variables were summarized using the mean and standard deviation or the median and interquartile range, as appropriate, whereas categorical variables were summarized using counts and percentages. All statistical analyses were performed by S.G.K., a PhD medical statistician and member of the investigative team. Analyses were conducted using Python 3.12.14 with scikit-learn 1.8.0, pandas 2.2.3, NumPy 2.3.5, SciPy 1.17.0, Matplotlib 3.10.8, and seaborn 0.13.2. Two-sided *p* values below 0.05 were considered statistically significant for the paired model comparisons.

Generative artificial intelligence (ChatGPT using GPT-5.5; OpenAI, San Francisco, CA, USA) was employed solely to improve the readability and linguistic presentation of the manuscript. Its use was limited to refining grammar, wording, and style and did not extend to data analysis, interpretation, study design, or scientific judgment. The authors independently developed and verified all scientific content and conclusions and accept full responsibility for the final manuscript.

## 3. Results

### 3.1. Study Population

Among 1180 patients screened, 905 were included in the final analytic cohort. Of these, 163 patients (18.0%) had a qualifying surgical procedure documented at the study institution within 90 days of the index ED visit, and 742 (82.0%) did not (Figure 1).

All 905 patients were included in each repeated cross-validation cycle. Within every repetition, each patient contributed one out-of-fold prediction, yielding 50 out-of-fold predictions per model and patient. Among the 163 patients with the study outcome, the mean time from the index ED visit to surgery was 4.4 ± 3.3 days, and the median was 3 days (interquartile range, 2–5 days). The observed range was 0–21 days, and 144 procedures (88.3%) were performed within 7 days. The distributions of recorded procedure categories and time to surgery are presented in Supplementary Table S6.

The baseline demographic, clinical, and radiographic characteristics of the study population according to whether surgery was documented at the study institution within 90 days are summarized descriptively in Table 1. No univariable hypothesis tests were conducted because these comparisons were not intended to support predictor-selection, independent-association, or causal inferences.

### 3.2. Model Performance

Across 50 repetitions of stratified five-fold cross-validation, the distributions of the repeat-level AUROC, AUPRC, and Brier score estimates are shown in Figure 2. Based on each patient’s average out-of-fold predicted probability, logistic regression achieved an AUROC of 0.762 (95% CI, 0.720–0.802), an AUPRC of 0.430 (95% CI, 0.372–0.507), and a Brier score of 0.125 (95% CI, 0.117–0.133). Random forest achieved an AUROC of 0.757 (95% CI, 0.712–0.802), an AUPRC of 0.443 (95% CI, 0.384–0.523), and a Brier score of 0.123 (95% CI, 0.116–0.130). The corresponding estimates for gradient-boosted trees were 0.747 (95% CI, 0.704–0.791), 0.395 (95% CI, 0.340–0.468), and 0.130 (95% CI, 0.121–0.140), respectively (Table 2).

Paired bootstrap comparisons did not identify statistically significant differences between random forest and logistic regression in AUROC, AUPRC, or Brier score. Compared with gradient-boosted trees, random forest had a higher AUPRC (difference, 0.048; 95% CI, 0.009–0.090; *p* = 0.017) and a lower Brier score (difference, −0.007; 95% CI, −0.011 to −0.003; *p* = 0.002), whereas the AUROC difference was not statistically significant. Complete paired comparisons are presented in Supplementary Table S4. Across the 50 repeated cross-validation cycles, logistic regression ranked first for AUROC in 41 cycles (82.0%), whereas random forest ranked first for AUPRC in 47 cycles (94.0%) and for Brier score in 46 cycles (92.0%) (Supplementary Table S7). Cross-validated receiver operating characteristic and precision–recall curves based on the patient-level average out-of-fold predictions are presented in Supplementary Figure S1.

### 3.3. Calibration Performance

Calibration based on patient-level average out-of-fold predictions is shown in Figure 3. Logistic regression had a calibration-in-the-large value of 0.000 (95% CI, −0.074 to 0.076) and a calibration slope of 0.939 (95% CI, 0.766–1.146). Random forest had a calibration-in-the-large value of −0.009 (95% CI, −0.072 to 0.052) and a calibration slope of 1.099 (95% CI, 0.883–1.357). Gradient-boosted trees had a calibration-in-the-large value of 0.054 (95% CI, −0.037 to 0.144) and a calibration slope of 0.758 (95% CI, 0.608–0.927). Calibration-group counts, event numbers, mean predicted probabilities, and observed outcome proportions are reported in Supplementary Table S5.

### 3.4. Model Interpretability

Validation-fold permutation analysis ranked knee swelling, limited ROM, and injury mechanism as the three most influential random-forest predictors. Permuting these variables reduced validation-fold AUPRC by a mean of 0.146, 0.110, and 0.106, respectively (Table 3 and Figure 4). The remaining predictors produced substantially smaller mean changes in AUPRC when permuted. Because permutation importance measures the loss of predictive performance after predictor information is disrupted, these results do not indicate the direction, independence, or causality of associations.

## 4. Discussion

In this secondary analysis of the cohort used in our previous study, we evaluated the robustness of model performance and predictor-importance patterns using 50 repetitions of stratified five-fold cross-validation. Logistic regression yielded the highest AUROC, whereas random forest yielded the highest AUPRC and lowest Brier score; however, paired bootstrap comparisons did not identify statistically significant differences between these two models for any of the three performance measures. Random forest showed a significantly higher AUPRC and lower Brier score than gradient-boosted trees, although their AUROCs did not differ significantly. Validation-fold permutation importance identified knee swelling, limited range of motion, and injury mechanism as the most influential random-forest predictors. Overall, repeated internal validation did not identify a uniformly superior model. These findings represent internal estimates from a single-center cohort and require temporal and external validation before clinical application.

Validation-fold permutation importance identified knee swelling, limited ROM, and injury mechanism as the strongest contributors to random-forest predictive performance. Swelling and ROM restriction are clinically relevant components of the assessment of acute knee trauma [20], but they are nonspecific findings. They may act as proxies for the severity of an underlying structural injury, joint effusion or hemarthrosis, pain-related motion restriction, or mechanical obstruction rather than having independent biological relationships with subsequent surgery. Their recorded presence may also reflect variation in clinical assessment and ED documentation. Similarly, injury mechanism may capture differences in the patterns and severity of underlying structural injury. Because permutation importance is conditional on the fitted model and the joint distribution of the predictors, these findings should not be interpreted as independent effects, monotonic associations, causal relationships, or stand-alone indications for surgery.

Random forest was selected for the targeted interpretability analysis to evaluate the robustness of predictor-importance patterns for a nonlinear model and to maintain continuity with our previous analysis, rather than because it was statistically superior. Paired comparisons did not establish superiority of random forest over logistic regression. The permutation-importance findings are therefore specific to the fitted random forest and should not be interpreted as demonstrating that the same predictor ranking would be obtained from logistic regression or gradient-boosted trees.

Previous machine learning research in knee and orthopedic trauma has included both model-development and validation studies. For example, a model-development study evaluated the 2- and 5-year risk of total knee arthroplasty after tibial plateau fracture using internal training and test samples [10]. Systematic reviews of models for outcomes after anterior cruciate ligament reconstruction and orthopedic trauma have shown that most studies focused on model development and internal validation, whereas evaluations of previously developed models in independent populations were relatively uncommon [9,11]. This distinction is important because internal validation estimates reproducibility within the development population, whereas external validation evaluates transportability to different settings, time periods, or patient populations. The present study is neither external validation nor independent model development in a new cohort; it is an exploratory robustness analysis applying repeated internal validation to the cohort used in our previous study. Therefore, the revised analysis provides more stable estimates of internal performance and resampling variability but does not establish external generalizability.

The distribution of recorded procedures further illustrates the clinical heterogeneity of the study outcome (Supplementary Table S6). The recorded procedures included arthroscopy, fracture fixation, meniscal procedures, ligament reconstruction or repair, external fixation, arthroplasty, and staged procedures, which have different indications and treatment timelines. For example, a fracture requiring urgent fixation and a meniscal lesion treated arthroscopically at a later time represent clinically distinct events, although both were classified as having the study outcome. The median time to surgery was 3 days, 88.3% of procedures were performed within 7 days, and the observed range was 0–21 days. Nevertheless, the outcome window was prespecified as 90 days to allow the capture of delayed procedures. The models should therefore be interpreted as predicting a heterogeneous institutional treatment pathway rather than a uniform diagnosis, procedure, or biological need for surgery.

Surgery documented within 90 days should also be understood as the result of a clinical treatment pathway. Many acute knee injuries are initially managed with analgesia, activity modification, immobilization or bracing when appropriate, physiotherapy or rehabilitation, and subsequent specialist reassessment [2]. Patellar instability or dislocation provides a specific example of this heterogeneity because both conservative and surgical strategies may be used, and restoration of quadriceps strength and function represents an important component of recovery after primary or recurrent patellar dislocation [21]. The transition from initial non-operative management to surgery may be influenced by the injury pattern and severity, response to conservative treatment, clinician preference, patient preference, healthcare access, and institutional practice. Consequently, the study outcome reflects observed institutional treatment behavior rather than a purely biological outcome or an objective determination that surgery was necessary.

The present findings should also be interpreted in relation to our previous analysis of the same cohort [17]. In that study, logistic regression and random forest showed similar discrimination, whereas XGBoost yielded lower performance estimates. The current repeated-validation analysis again demonstrated closely matched performance between logistic regression and random forest, while the gradient-boosted tree model performed less favorably. Because the gradient-boosting implementation used in the present study was not identical to XGBoost, the third-model results should not be interpreted as a direct algorithm-to-algorithm replication. Moreover, absolute performance estimates should not be directly compared between the two studies because different internal-validation frameworks were used. The principal contribution of the present analysis is the explicit quantification of resampling variability, paired model differences, quantitative calibration, and model-rank stability within the overlapping cohort.

Across repeated internal validation, logistic regression yielded the highest AUROC, whereas random forest yielded the highest AUPRC and lowest Brier score. However, paired bootstrap comparisons did not identify statistically significant differences between these two models for any of the three performance measures. This finding is consistent with previous evidence that machine learning algorithms do not uniformly outperform logistic regression in clinical prediction studies [12]. Random forest showed a significantly higher AUPRC and lower Brier score than gradient-boosted trees, but this result should not be interpreted as evidence that gradient boosting is generally inferior. The comparatively less favorable performance of gradient-boosted trees may have been specific to the present cohort, limited predictor set, fixed modeling configuration, or other unmeasured features of the analysis; these potential explanations were not evaluated directly.

The sensitivity of absolute performance estimates to the composition of a single training–test partition indicates that reliance on one random split was suboptimal and that repeated resampling would have been preferable from the outset. We therefore replaced the single alternative holdout partition with 50 repetitions of stratified five-fold cross-validation. This approach used all available patients for both model fitting and out-of-fold evaluation across repeated cycles and allowed resampling variability and model-rank stability to be quantified. Nevertheless, performance estimates and model rankings continued to vary across repetitions and according to the evaluation metric, indicating residual model instability and the absence of a uniformly superior algorithm. Repeated internal validation reduces dependence on a particular partition but cannot eliminate optimism or establish transportability. Temporal validation and evaluation in independent institutions therefore remain necessary [13,14,22].

The models used variables routinely available during the initial ED assessment, allowing us to evaluate whether these data contained predictive information before advanced imaging or subsequent orthopedic decision-making. However, the present findings were derived from retrospective internal validation at a single institution, and the outcome represented a heterogeneous set of qualifying surgical procedures documented at that institution rather than an objective indication for surgery. Therefore, no specific clinical role can be inferred from the present analysis. The models should not be used to prioritize orthopedic consultation, determine follow-up intensity, or guide surgical decision-making until their transportability, calibration, and clinical utility have been established through external validation and prospective evaluation.

Quantitative and graphical calibration assessment showed calibration-in-the-large values close to 0 for all three models, with confidence intervals that included the ideal value. The calibration slopes for logistic regression and random forest were also compatible with the ideal value of 1. In contrast, the calibration slope for gradient-boosted trees was below 1, indicating that its predicted probabilities were overly extreme in this cohort. These estimates were derived entirely from internal resampling and therefore require reassessment and, if necessary, recalibration in independent populations [23].

Decision-curve analysis was not repeated because the present secondary analysis focused on the robustness of discrimination, precision–recall performance, probabilistic accuracy, calibration, and model-rank stability under repeated internal validation. Threshold-specific performance and decision-curve analyses using the same underlying cohort were reported previously [17]. However, those findings were also derived from the same single-center cohort and should not be interpreted as establishing clinical net benefit in other settings. Before clinical implementation, the models would require temporal and external validation, recalibration where necessary, and prospective assessment of whether their use improves patient management or outcomes compared with usual clinical judgment.

This study has several limitations. First, this was a retrospective study conducted at a single institution, which limits transportability to other hospitals, patient populations, and clinical settings. Repeated cross-validation improves the stability of internal performance estimates but does not replace temporal or external validation. The cohort size was fixed by the available retrospective data, and no formal prospective sample-size calculation was performed. Although the models used a limited predictor set, constrained complexity, and repeated internal validation, these measures do not eliminate the possibility of overfitting or instability, particularly for the tree-based models. Second, 128 patients (10.8% of those screened) were excluded because of missing predictor data and/or insufficient institutional follow-up to ascertain the study outcome. Because these exclusion mechanisms were retained only as a combined category and individual-level data for the excluded patients were unavailable, we could neither quantify the two mechanisms separately, calculate predictor-specific missingness among all screened patients, nor compare the baseline characteristics of excluded and included patients. Multiple imputation could not be performed retrospectively because the incomplete individual-level records and predictor-specific missingness patterns were unavailable; moreover, predictor imputation would not have resolved the absence of outcome ascertainment in patients with insufficient follow-up. Consequently, the direction and magnitude of potential selection bias could not be assessed. Third, a major limitation concerns both the ascertainment and clinical heterogeneity of the study outcome. The endpoint was limited to qualifying surgical procedures documented at the study institution within 90 days. Surgical procedures performed at other institutions could not be identified, and some patients who underwent surgery elsewhere may therefore have been misclassified as not having the study outcome. The dataset also lacked a structured indicator of an additional knee trauma during the 90-day follow-up period. We therefore could not exclude the possibility that a subsequent injury contributed to some recorded procedures, creating an additional potential source of outcome misclassification. Furthermore, the endpoint combined clinically distinct procedures with different diagnoses, indications, and degrees of urgency. For example, urgent fracture fixation and a later arthroscopic meniscal procedure were classified as the same outcome despite representing substantially different clinical events. Although the recorded procedure categories and timing are reported in Supplementary Table S6, a separate structured variable for the injury-specific operative diagnosis was unavailable, and the broad procedure categories did not permit procedure-specific prediction. The model output therefore reflects observed institutional treatment behavior and should not be interpreted as predicting a particular operation, an objective biological indication for surgery, or the necessity of surgical treatment. Fourth, the models were based on variables available during the initial ED assessment and did not include potentially relevant information such as advanced imaging findings, detailed physical examination findings, injury-specific diagnoses, comorbidities, functional status, or subsequent clinical course. In addition, knee swelling and ROM limitation were retrospectively abstracted from routine ED documentation without quantitative measurements or grading thresholds. Although disagreements between the two reviewers were resolved by consensus, their initial individual ratings were not retained, precluding calculation of interobserver agreement. These variables may therefore have been affected by subjective assessment and variability in clinical documentation. Although excluding information obtained after the index assessment reduced the risk of temporal leakage, the limited predictor set may have constrained model performance. Fifth, permutation importance was restricted to the random forest model and is conditional on the fitted algorithm and predictor distribution. It does not describe the direction or independent effect of a predictor, and the resulting importance pattern may not generalize to logistic regression, gradient-boosted trees, or external populations. Finally, although calibration and paired model differences were assessed quantitatively, these estimates were derived entirely from internal resampling of a single-center cohort. Clinical utility was not evaluated using decision-curve analysis or prospective impact assessment. External and temporal validation, recalibration where necessary, and evaluation of clinical usefulness are required before implementation.

## 5. Conclusions

In this secondary analysis of a previously reported cohort, repeated internal validation showed no uniformly superior prediction model. Logistic regression most often ranked first for AUROC, whereas random forest most often ranked first for AUPRC and Brier score; however, paired comparisons did not identify significant differences between these two models. The relative performance pattern was broadly consistent with that observed in our previous analysis, but the present findings remain a sensitivity and robustness analysis of the same cohort rather than independent model development or external validation. The study does not validate a clinically deployable prediction tool, and clinical utility has not been established. Temporal and external validation, recalibration where necessary, and prospective assessment of clinical usefulness are required before implementation.

## Figures and Tables

**Figure 1:**
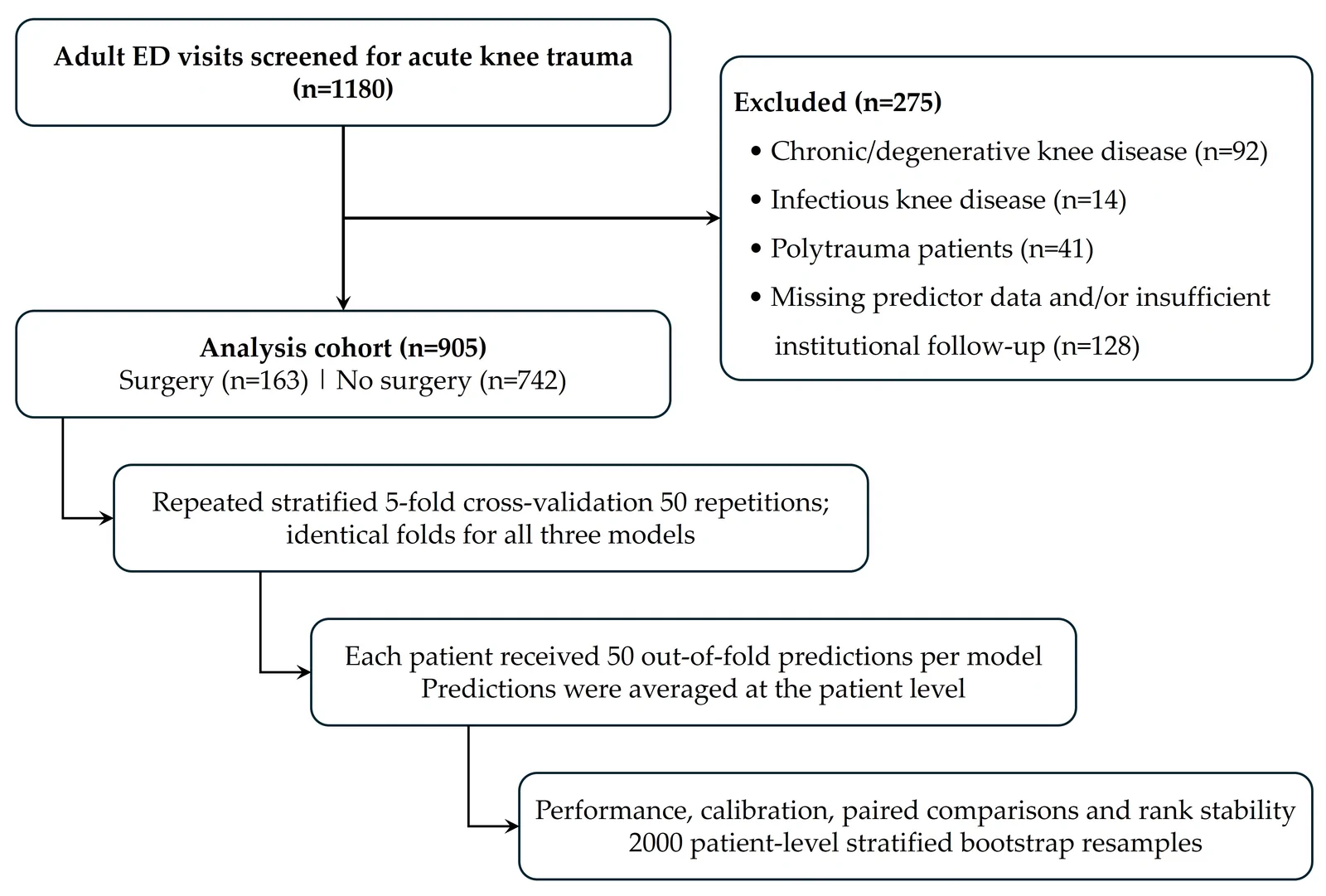
Flowchart of cohort construction and repeated internal validation. Repeated stratified five-fold cross-validation produced 50 out-of-fold predictions per patient and model; patient-level average predictions were used for performance and calibration analyses.

**Figure 2:**
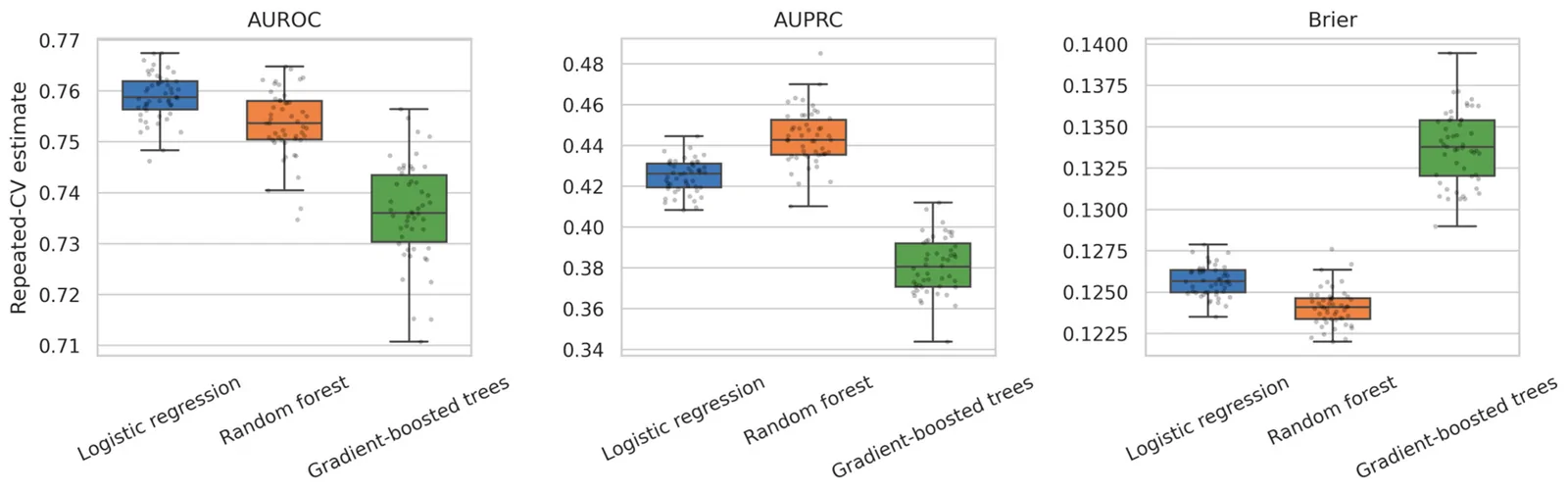
Distribution of AUROC, AUPRC, and Brier score estimates across 50 repetitions of stratified five-fold cross-validation. Boxes show the interquartile range with the median; whiskers and individual repeat-level estimates show between-repetition variability. Higher AUROC and AUPRC and lower Brier scores indicate better performance.

**Figure 3:**
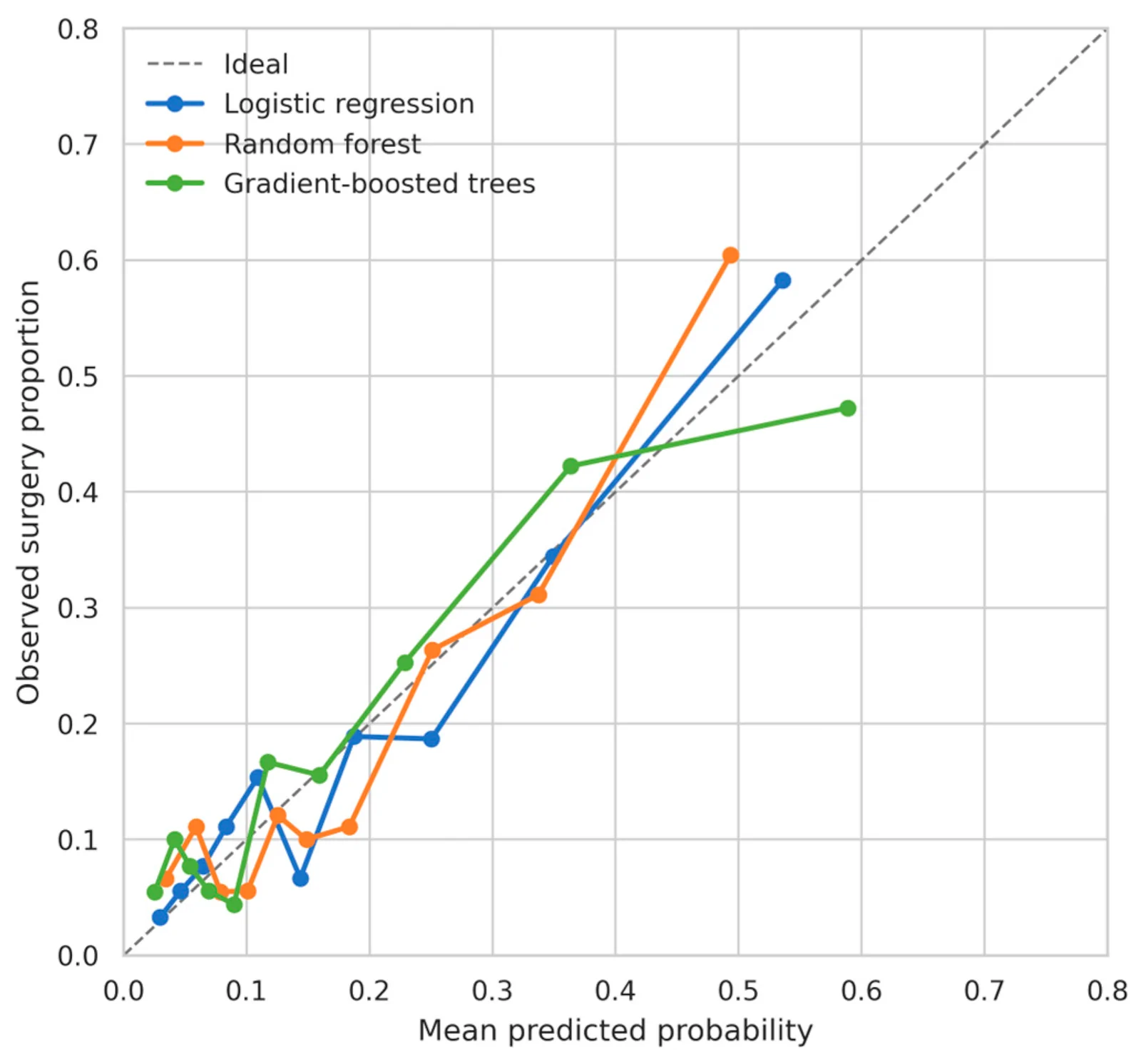
Cross-validated calibration plots based on patient-level average out-of-fold predicted probabilities. Points represent 10 equal-frequency groups and compare the mean predicted probability with the observed proportion of patients with surgery documented at the study institution within 90 days. The dashed diagonal denotes ideal calibration.

**Figure 4:**
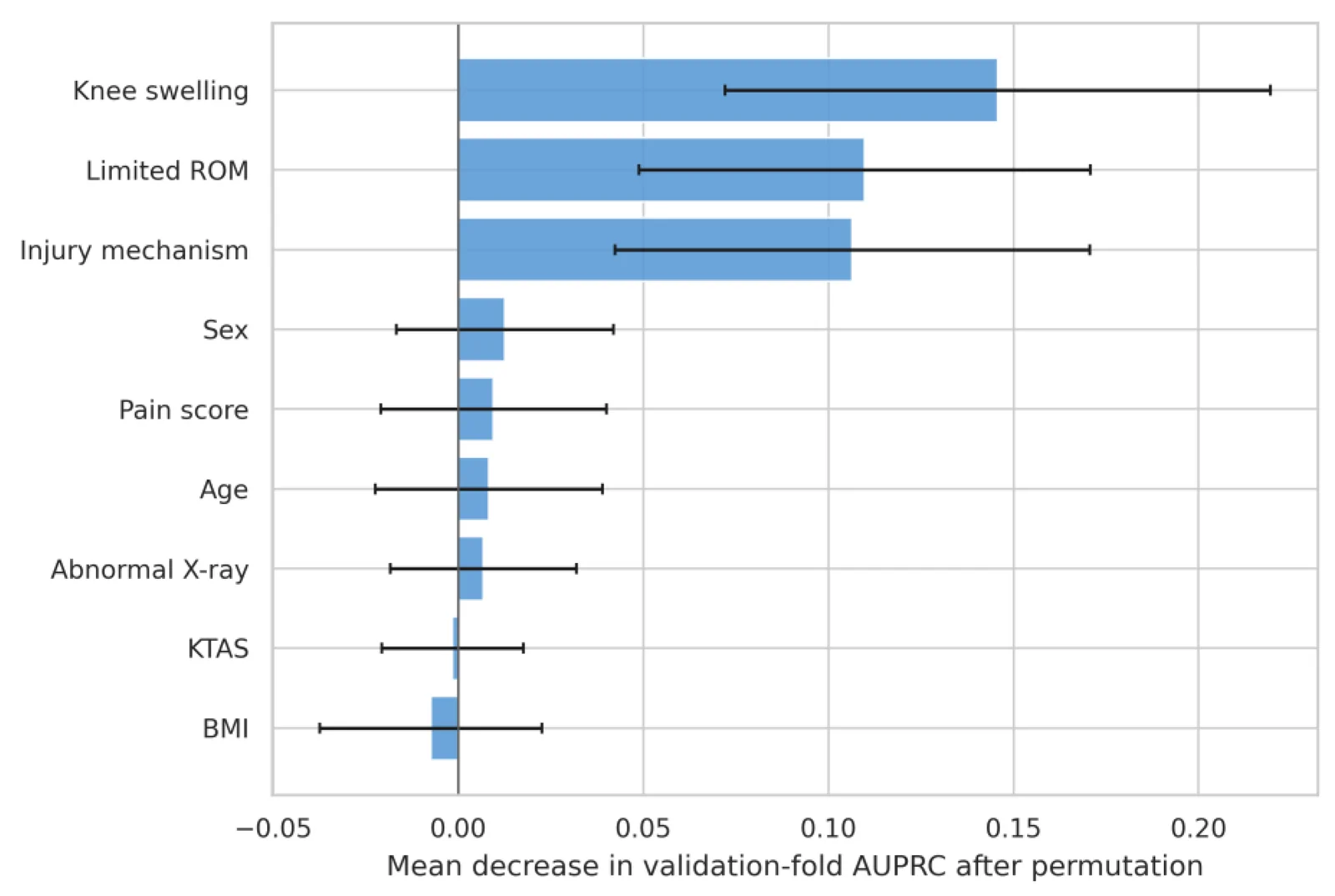
Random-forest validation-fold permutation importance. Bars represent the mean decrease in AUPRC after permutation of each original predictor, and error bars represent the standard deviation across 50 validation folds and three permutations per predictor. Larger positive values indicate a greater contribution to predictive performance.

**Table 1:** Baseline characteristics according to whether surgery was documented at the study institution within 90 days.

Variable		No Documented Surgery (*n* = 742)	Documented Surgery (*n* = 163)
Age, years		48.0 ± 19.0	52.9 ± 20.0
Sex	Male	443 (59.7)	109 (66.9)
	Female	299 (40.3)	54 (33.1)
BMI, kg/m^2^		25.4 ± 3.8	24.8 ± 4.1
Injury mechanism	Fall	266 (35.8)	41 (25.2)
	Sports injury	216 (29.1)	58 (35.6)
	Twisting	152 (20.5)	17 (10.4)
	TA	46 (6.2)	35 (21.5)
	Direct blow	62 (8.4)	12 (7.4)
Pain score (NRS)		5.3 ± 1.5	5.9 ± 1.5
Knee swelling	No	492 (66.3)	54 (33.1)
	Yes	250 (33.7)	109 (66.9)
Limited ROM	No	509 (68.6)	67 (41.1)
	Yes	233 (31.4)	96 (58.9)
KTAS		3.9 ± 0.7	3.8 ± 0.8
Abnormal X-ray	No	629 (84.8)	115 (70.6)
	Yes	113 (15.2)	48 (29.4)

Values are presented as mean ± standard deviation or number (%). BMI, body mass index; NRS, numeric rating scale; KTAS, Korean Triage and Acuity Scale; ROM, range of motion; TA, traffic accident.

**Table 2:** Repeated cross-validation performance and calibration of the prediction models.

Model	AUROC	AUPRC	Brier Score	Calibration-in-the-Large	Calibration Slope
Logistic regression	0.762 (0.720–0.802)	0.430 (0.372–0.507)	0.125 (0.117–0.133)	0.000 (−0.074–0.076)	0.939 (0.766–1.146)
Random forest	0.757 (0.712–0.802)	0.443 (0.384–0.523)	0.123 (0.116–0.130)	−0.009 (−0.072–0.052)	1.099 (0.883–1.357)
Gradient-boosted trees	0.747 (0.704–0.791)	0.395 (0.340–0.468)	0.130 (0.121–0.140)	0.054 (−0.037–0.144)	0.758 (0.608–0.927)

Values are estimates with 95% confidence intervals calculated from patient-level average out-of-fold predictions using 2000 stratified patient-level bootstrap resamples. Ideal calibration corresponds to a calibration-in-the-large value of 0 and a calibration slope of 1. Lower Brier scores indicate better probabilistic accuracy. AUPRC, area under the precision–recall curve; AUROC, area under the receiver operating characteristic curve.

**Table 3:** Validation-fold permutation importance for the random forest model.

Rank	Predictor	Mean Decrease in AUROC (SD)	Mean Decrease in AUPRC (SD)
1	Knee swelling	0.083 (0.044)	0.146 (0.074)
2	Limited ROM	0.044 (0.029)	0.110 (0.061)
3	Injury mechanism	0.063 (0.035)	0.106 (0.064)
4	Sex	0.004 (0.009)	0.013 (0.029)
5	Pain score	0.001 (0.016)	0.010 (0.030)
6	Age	0.018 (0.023)	0.008 (0.031)
7	Abnormal X-ray	0.009 (0.017)	0.007 (0.025)
8	KTAS	0.002 (0.012)	−0.002 (0.019)
9	BMI	−0.002 (0.016)	−0.007 (0.030)

Importance was calculated in 50 validation folds from 10 repetitions of stratified five-fold cross-validation, with three permutations per predictor and fold. Predictors were ranked according to the mean decrease in AUPRC. Positive values indicate loss of validation performance after permutation; values near or below zero indicate little measurable contribution. AUPRC, area under the precision–recall curve; AUROC, area under the receiver operating characteristic curve; BMI, body mass index; KTAS, Korean Triage and Acuity Scale; ROM, range of motion; SD, standard deviation.

## Data Availability

The data presented in this study are available upon request from the corresponding author under ethical and legal restrictions related to patient confidentiality.

## Supplementary Materials

The following supporting information can be downloaded at: https://www.mdpi.com/article/10.3390/medicina62101821/s1, Table S1: Comparison of the previous publication and the present secondary analysis [17]; Table S2: Definitions, sources, and coding of predictor variables; Table S3: Model and validation settings; Table S4: Paired differences in model performance; Table S5: Equal-frequency calibration groups based on patient-level average out-of-fold predictions; Table S6: Recorded surgical procedure categories and time to surgery among 163 patients with surgery documented at the study institution within 90 days; Table S7: Model-rank stability across 50 repeated cross-validation cycles; Figure S1: Receiver operating characteristic and precision–recall curves based on patient-level average out-of-fold predictions from 50 repetitions of stratified five-fold cross-validation.

## Abbreviations

The following abbreviations are used in this manuscript:

AUPRC	Area under the precision–recall curve
AUROC	Area under the receiver operating characteristic curve
BMI	Body mass index
ED	Emergency department
KTAS	Korean Triage and Acuity Scale
NRS	Numeric rating scale
ROM	Range of motion
