# Supplementary material for: Repeated Internal Validation and Interpretation of Models Predicting Surgery Documented at a Study Institution Within 90 Days After Acute Knee Trauma: A Secondary Analysis
Source: Medicina (Kaunas). 2026 Sep 22;62(10):1821. doi: 10.3390/medicina62101821 (PMC13707078; doi:10.3390/medicina62101821)
Supplement: Supplementary file 1 [file medicina-62-01821-s001.zip › medicina-4516048-supplementary.pdf]

Supplementary materials

Table S1. Comparison of the previous publication and the present secondary analysis.

Component	Previous publication [17]	Present secondary analysis
Cohort	Same single-center cohort: 905 patients, including 163 with the outcome	Identical cohort; no new patients
Outcome	Surgical intervention within 90 days, ascertained from the study institution	Same underlying endpoint, explicitly termed "qualifying surgical procedures documented at the study institution within 90 days"
Candidate predictors	Nine variables available at the initial ED assessment	Same nine predictors; no claim of new predictor collection
Primary objective	Model development with emphasis on threshold-specific performance and clinical decision utility	Robustness/sensitivity analysis of performance, calibration, model ranking, and predictor importance under repeated internal resampling
Models	Logistic regression, random forest, and XGBoost	L2-regularized logistic regression, random forest, and gradient-boosted trees using fixed, constrained specifications
Primary internal validation	Single stratified 70:30 training-test split, with five-fold cross-validation in the training set	50 repetitions of stratified five-fold cross-validation, producing 50 out-of-fold predictions per patient and model
Performance measures	AUROC, AUPRC, and Brier score in cross-validation and the held-out test set	AUROC, AUPRC, and Brier score based on patient-level average out-of-fold predictions
Uncertainty estimation	Bootstrap confidence intervals for individual model performance in the held-out test set	Patient-level stratified bootstrap confidence intervals based on average out-of-fold predictions
Paired model comparisons	Not performed	Paired differences in AUROC, AUPRC, and Brier score using 2,000 patient-level stratified bootstrap resamples

Component	Previous publication [17]	Present secondary analysis
Model-rank stability	Not evaluated	Frequency and distribution of model rankings across 50 cross-validation repetitions
Calibration	Brier scores and calibration plots; quantitative calibration emphasized for the selected logistic model	Calibration-in-the-large and calibration slope with bootstrap confidence intervals for all three models, together with cross-validated calibration plots
Interpretability	SHAP-based predictor-attribution analysis	Validation-fold permutation importance for random forest, evaluated across 50 validation folds with repeated permutations
Threshold-specific analysis	Sensitivity, specificity, PPV, and NPV across selected thresholds	Not repeated; findings are explicitly attributed to the previous publication
Decision-curve analysis	Performed	Not repeated; findings are explicitly attributed to the previous publication
Risk stratification	Low-, intermediate-, and high-risk groups evaluated	Not repeated
Temporal validation	Logistic regression evaluated in a temporally separated cohort	Not repeated and not presented as a new result
Clinical characterization of outcome	Procedure-category distribution and timing not reported in detail	Recorded procedure categories and time to surgery reported in Supplementary Table S6
Reuse of published material	Not applicable	No previously published tables or figures reused
Intended interpretation	Model development and exploratory clinical decision utility	Exploratory robustness/sensitivity analysis; not an independent validation study or clinically deployable tool

Table S2. Definitions, sources, and coding of predictor variables.

Predictor	Operational definition and source	Recorded values	Model representation
Age	Age at index ED visit	Years	Continuous
Sex	Recorded biological sex	Male<br>Female	Binary
BMI	Weight (kg)/height $(m)^2$	kg/m^2	Continuous
Injury mechanism	Documented mechanism of injury	Fall<br>Sports<br>Twisting<br>Traffic accident<br>Direct blow	Dummy variables
Pain score	Initial NRS pain score	0–10	Continuous
Knee swelling	Explicit documentation of swelling, effusion, or joint effusion of the injured knee during the initial ED physical examination	No<br>Yes	Binary (0/1)
Limited ROM	Explicit documentation of limited or decreased knee ROM, restricted flexion or extension, pain-limited motion, or mechanical block during the initial ED examination	No<br>Yes	Binary (0/1)
KTAS	Triage acuity level	1–5*	Ordinal numeric
Abnormal X-ray	Acute traumatic abnormality on plain radiography†	No<br>yes	Binary (0/1)

All predictors were obtained during the index ED assessment. Advanced imaging findings, orthopedic treatment decisions, and information recorded after the initial assessment were not used. Knee swelling and ROM limitation were binary variables abstracted from routine ED documentation and were not based on quantitative measurements or grading thresholds. All nine candidate predictors had 0% missingness in the final analytic cohort by construction. Predictor-specific missingness among all screened patients could not be reconstructed because the excluded patients were retained only as a combined exclusion category in the available de-identified dataset. BMI, body mass index; ED, emergency department; KTAS, Korean Triage and Acuity Scale; NRS, numeric rating scale; ROM, range of motion. * Lower KTAS values indicate greater urgency. † Includes fracture, dislocation, or another acute traumatic osseous or joint abnormality.

Table S3. Model and validation settings.

Component	Setting	Value
Logistic regression	Penalty	L2
	Inverse regularization strength	1.0
	Solver / maximum iterations	lbfgs / 2,000
Random forest	Number of trees	500
	Maximum depth	10
	Minimum samples per leaf	4
	Features considered per split	Square root
Gradient-boosted trees	Number of estimators	300
	Learning rate	0.03
	Maximum depth	3
	Minimum samples per leaf	4
	Row subsampling	0.9
	Feature subsampling	0.9
All models	Class weighting	None
Validation	Resampling	Stratified 5-fold CV × 50 repetitions
	Fold assignment	Identical across models
	Primary prediction	Mean of 50 OOF probabilities per patient
Uncertainty	Bootstrap	2,000 patient-level stratified resamples
Reproducibility	Primary random seed	20260912

Hyperparameters were fixed before the repeated-validation analysis. Preprocessing was fitted separately within each training fold. CV, cross-validation; OOF, out-of-fold.

Table S4. Paired differences in model performance.

Comparison	Metric	Difference (95% CI)	p value
Random forest minus Logistic regression	AUROC	-0.005 (-0.026 to 0.016)	0.604
	AUPRC	0.012 (-0.032 to 0.062)	0.604
	Brier	-0.002 (-0.006 to 0.002)	0.372
Gradient-boosted trees minus Logistic regression	AUROC	-0.014 (-0.039 to 0.012)	0.244
	AUPRC	-0.036 (-0.086 to 0.013)	0.156
	Brier	0.005 (-0.001 to 0.011)	0.081
Random forest minus Gradient-boosted trees	AUROC	0.009 (-0.009 to 0.027)	0.320
	AUPRC	0.048 (0.009 to 0.090)	0.017
	Brier	-0.007 (-0.011 to -0.003)	0.002

Differences are first model minus second model and are based on the same 2,000 patient-level stratified bootstrap samples. For AUROC and AUPRC, positive differences favor the first model; for Brier score, negative differences favor the first model. CI, confidence interval.

Table S5. Equal-frequency calibration groups based on patient-level average out-of-fold predictions.

Model	Group	n	Events	Mean predicted	Observed
LR	1	91	3	0.030	0.033
	2	90	5	0.046	0.056
	3	91	7	0.065	0.077
	4	90	10	0.083	0.111
	5	91	14	0.109	0.154
	6	90	6	0.144	0.067
	7	90	17	0.187	0.189
	8	91	17	0.250	0.187
	9	90	31	0.349	0.344
	10	91	53	0.536	0.582
RF	1	91	6	0.034	0.066
	2	90	10	0.059	0.111
	3	91	5	0.079	0.055
	4	90	5	0.101	0.056
	5	91	11	0.125	0.121
	6	90	9	0.149	0.100
	7	90	10	0.184	0.111
	8	91	24	0.251	0.264
	9	90	28	0.337	0.311
	10	91	55	0.494	0.604
GBT	1	91	5	0.025	0.055
	2	90	9	0.041	0.100
	3	91	7	0.054	0.077
	4	90	5	0.069	0.056
	5	91	4	0.090	0.044
	6	90	15	0.117	0.167
	7	90	14	0.159	0.156
	8	91	23	0.229	0.253
	9	90	38	0.364	0.422
	10	91	43	0.589	0.473

Groups were defined separately for each model using deciles of the average out-of-fold predicted probabilities. Observed values are the proportions undergoing surgery within 90 days at the study institution.

Table S6. Recorded surgical procedure categories and time to surgery among 163 patients with surgery documented at the study institution within 90 days.

Section	Item	Value
Procedure type	Arthroscopy	45 (27.6%)
	ORIF	42 (25.8%)
	Meniscus repair	26 (16.0%)
	ACL/PCL reconstruction	18 (11.0%)
	Partial meniscectomy	10 (6.1%)
	Collateral/Multiligament repair	8 (4.9%)
	External fixation	5 (3.1%)
	Multiligament reconstruction	5 (3.1%)
	Arthroplasty	2 (1.2%)
	Staged procedure	2 (1.2%)
Timing	Time to surgery, days, mean ± SD	4.4 ± 3.3
	Time to surgery, days, median (IQR)	3 (2–5)
	Time to surgery, days, range	0–21
	Surgery on day 0–1	26 (16.0%)
	Surgery within 7 days	144 (88.3%)

Procedure categories are mutually exclusive and are presented as recorded in the analytic dataset. Percentages for procedure categories were calculated using the 163 patients with the study outcome as the denominator. Time to surgery was measured from the index ED visit. A separate structured variable for the injury-specific operative diagnosis was unavailable. No cases were recorded under separate categories for patellar procedures or extensor-mechanism repair. ACL, anterior cruciate ligament; IQR, interquartile range; ORIF, open reduction and internal fixation; PCL, posterior cruciate ligament.

Table S7. Model-rank stability across 50 repeated cross-validation cycles.

Metric	Model	Ranked first, n/50	Ranked first, %	Median rank
AUROC	LR	41/50	82.0	1
	RF	9/50	18.0	2
	GBT	0/50	0.0	3
AUPRC	LR	3/50	6.0	2
	RF	47/50	94.0	1
	GBT	0/50	0.0	3
Brier	LR	4/50	8.0	2
	RF	46/50	92.0	1
	GBT	0/50	0.0	3

First rank was assigned within each repeated cross-validation cycle. Lower Brier score and higher AUROC/AUPRC were ranked better. AUPRC, area under the precision–recall curve; AUROC, area under the receiver operating characteristic curve; GBT, gradient-boosted trees; LR, logistic regression; RF, random forest.

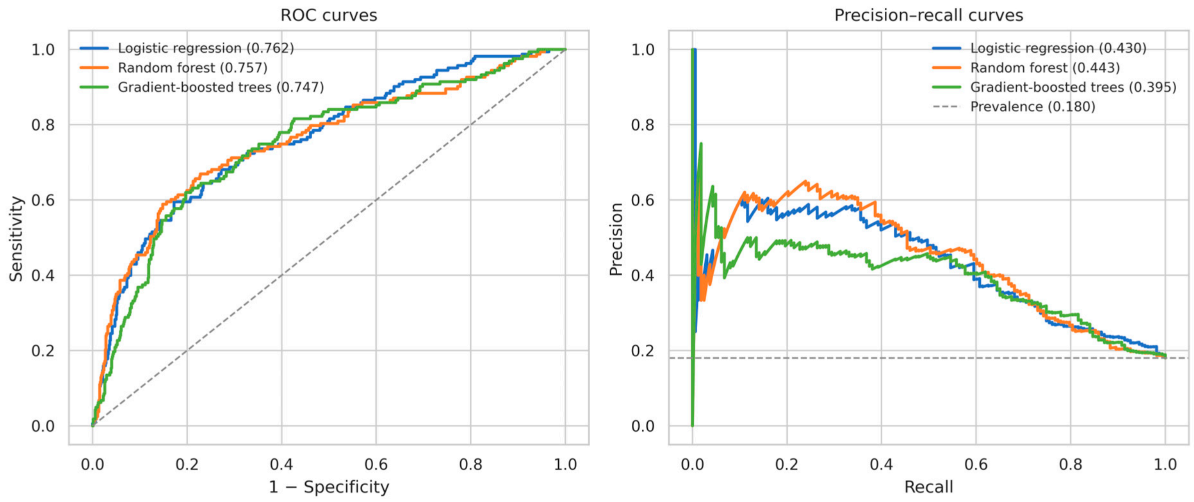

Figure S1. Receiver operating characteristic and precision–recall curves based on patient-level average out-of-fold predictions from 50 repetitions of stratified five-fold cross-validation. Legends report AUROC and AUPRC, respectively; the dashed horizontal line in the precision–recall panel indicates the outcome prevalence (0.180).
